# Independent or Influential? Spatial-Temporal Features of Coordination Level between Urbanization Quality and Urbanization Scale in China and Its Driving Mechanism

**DOI:** 10.3390/ijerph17051587

**Published:** 2020-03-01

**Authors:** Yijun Shi, Quan Zhu, Lihua Xu, Zhangwei Lu, Yaqi Wu, Xiangbin Wang, Yang Fei, Jinyang Deng

**Affiliations:** 1 School of Landscape Architecture, Zhejiang A&F University, Hangzhou 311300, China; zhuquan@zafu.edu.cn (Q.Z.); xulihua@zafu.edu.cn (L.X.); zhwlu@zafu.edu.cn (Z.L.); 20030050@zafu.edu.cn (Y.W.); 16621351725@163.com (X.W.); fei___yang@163.com (Y.F.); 2 School of Natural Resources, West Virginia University, Morgantown, WV 26506, USA; jinyang.deng@mail.wvu.edu

**Keywords:** urbanization coordination level, urbanization quality, urbanization scale, spatial-temporal features, driving mechanism

## Abstract

The quality and scale of urbanization development are the two main aspects in China’s current urbanization process. By measuring and analyzing the level differences in these two aspects, the healthy development of China’s urbanization and urban–rural integration will be promoted. Based on the quality of urbanization and the scale of urbanization, this paper constructs an evaluation index system for urbanization coordination level. On this basis, this paper analyzes the spatial correlation, spatial difference, and spatial pattern evolution characteristics of urbanization coordination level in 286 sample cities nationwide from 2005 to 2015. Then, by introducing the spatial econometric regression model, this paper discusses the driving mechanism of the spatial and temporal evolution of urbanization coordination level. The results show that: (1) The level of coordination between urbanization quality and urbanization scale shows a strong spatial correlation in space, which is consistent with the actual development status; (2) the level of urbanization coordination shows a trend of evolution from northeast to southwest in the evolution of spatial pattern, but the extent of change is small; and (3) the spatial and temporal pattern of urbanization coordination level is affected by different driving forces, of which internal source is the primary impact factor, followed by administrative level and investment level. In addition, the level of urbanization coordination has a positive spillover effect on the level of urbanization coordination in adjacent areas.

## 1. Introduction

With the implementation of the reform and opening policy in 1978, China has been experiencing rapid urbanization and industrialization at an unprecedented rate. Since 1996, China’s urbanization has entered an accelerated stage of rapid development, whereas its urbanization rate has exceeded 30%, and maintained an average annual growth rate of 1.02%. In the process of China’s current urbanization development: (1) On the one hand, a large number of rural populations are pouring into cities and towns, and the scale of rural areas is shrinking while the scale of cities and towns is constantly expanding. This stage is the primary stage of urbanization development. We call it the stage of “quantity” expansion in the process of urbanization. (2) On the other hand, urban areas are gradually modernizing. We call it the stage of “quality” ascension in the process of urbanization. It should be noted that these two aspects have not been prioritized in China’s urbanization process, but have been carried out simultaneously [1,2]. Recognizing that the process of healthy urbanization should be a coordinated process of quantitative expansion and qualitative improvement, has important guiding significance for China’s current urbanization development, especially at this stage of rapid urban development. This is because China has just entered the stage of rapid urbanization. In the face of the endless stream of increasingly serious urban problems, it is impossible to use a large amount of funds for governance after entering the mature period of urbanization, as the European and American countries. It is also impossible to deal with various types of urban problems such as the third world countries [3]. The process of urbanization is not a short-term process, but a historical process of the transformation of human production and lifestyle from rural to urban. It is not only manifested in the transformation of rural population to urban population, the increase of urban towns, and the expansion of urban land. It can be said that the current development process of urbanization in China is a development process that involves the continuous expansion of urban scale and the continuous improvement of the quality of urbanization development [4].

In the process of urbanization development, due to the influence of the level of social and economic development, the development of urbanization will continue to change. In this process, due to the restrictions of the city’s administrative boundaries and urban land, the urban scale will not grow infinitely, and it will maintain a stable level after reaching a certain scale. Therefore, the scale of urbanization development will not increase indefinitely, it will stabilize after reaching a certain scale, and the growth rate will not be obvious, especially the scale performance of urban land is the most obvious [5]. However, the elements of the quality of urbanization development, such as socio-economic level, cultural, and scientific and technological innovation, can theoretically increase indefinitely. Studies by some scholars also show that during a certain period of urbanization development, there is a positive correlation between the scale of urbanization and the quality of urbanization [1,6,7,8,9,10]. For the current urbanization process in China, there is an interaction mechanism between the quality of urbanization and the scale of urbanization. The coordinated development between them is the key to continuously improve the level of urbanization coordination. By measuring and analyzing the level of urbanization coordination, it is helpful to recognize and understand the differences between different regions in China’s current urbanization development process, which is conducive to the healthy development of urbanization and urban-rural coordination for China [8,9,10].

The problem of coordination of urbanization, also called sustainability, has always been the focus of scholars. Existing studies have been conducted from the perspective of whether industrialization, population, land industry, and other aspects are coordinated with the development of urbanization. Existing research can be summarized into two categories: Qualitative research and quantitative research. In terms of the former, scholars have mainly discussed from the perspective of urban and rural labor mobility, resource and environmental foundations, and coordinated development of population and land urbanization. Gu et al. studied the coordinated development of urbanization and industrialization in China from the perspective of urban and rural labor mobility, and proposed a series of measures to promote the coordinated development of industrialization and urbanization, including: Encouraging the return of migrant workers and promoting civilization and promotion labor market integration and equal treatment, development of metropolitan areas, and development of rural urbanization centered on counties [11]. Yao et al. analyzed whether the speed and scale of urbanization in China were coordinated from the perspective of resource and environmental foundation [12]. Chen et al. analyzed the current status of urbanization in China from the perspective of coordinated development of population and land urbanization. It also gives specific suggestions on improving the coordination of population urbanization and land urbanization in terms of promoting the civilization of migrant workers and guiding the population to small and medium cities [13]. In the quantitative analysis, the methods of quadrant diagram, coupling degree model, Theil coefficient model, and spatial analysis method are mainly used to analyze urbanization coordination of related elements. Li et al. evaluated the coordination between urbanization and industrialization in various regions of China by using the ratio between the proportion of urban population and changes in the share of agricultural labor [14]. Cao et al. studied the coordination of population, land, and economic urbanization from the perspective of system theory [15]. Zhang et al. carried out a quantitative study on the coordination of the quality and scale of urbanization in Jiangsu Province by using the quadrant method [16]. Li et al. used a coupling model to study the coordination of urbanization in Chongqing from three dimensions: Land, population, and industry [17]. Li et al. used the Theil coefficient to measure the coordination of 105 prefecture-level urbanizations in the Yangtze River Economic Belt from four aspects: Urban and rural, industrial cities, regions and urbanization and resources and environment [18]. However, since urbanization itself is a complex system and coordination is a comprehensive concept, the research results of different scholars are not completely consistent. For example, Chang et al. analyzed the ratio relationship between China’s per capita income and urbanization since 1978, and argued that the rapid economic development in recent years has led to the lag of urbanization [4]. Chen et al. found that from 1960 to 2010, the level of urbanization in China was coordinated with economic development as a whole, and the rapid development after 2004 was closely related to the speed of economic development [19]. Li et al. analyzed the relationship between the urbanization level and ecological environment of Lianyungang City from 2000 to 2008, and found that the degree of coupling and coordination between urbanization level and ecological environment was U-shaped curve with time [20]. Wang et al. found that the degree of coupling and coordination between the urbanization of Beijing-Tianjin-Hebei population and the ecological environment showed an S-shaped curve over time [21]. Ma et al. analyzed the development coordination degree of each city in the middle reaches of the Yangtze River from three aspects of economic, social and ecological space, and analyzed its regional differences [22].

To sum up, most of the existing researches focus on one aspect of system elements in the process of urbanization from a certain perspective. The quality and scale of urbanization are the two most important aspects in the process of urbanization, and the research on their coordination is still scarce. Although some scholars have noticed this and tried to explore the relationship between the scale and quality of urbanization, qualitative research is still the main focus. As for quantitative studies, most have adopted the quadrant diagram method. On the one hand, this method is subjective in dividing the type of coordination relationship between the quality and scale of urbanization, so as to affect the results of the study. On the other hand, there is a lack of research on the spatiotemporal features of urbanization quality and the scale of urbanization and their driving mechanisms [2]. Based on this perspective, this paper proposes quantitative methods for measuring the level of urban quality and scale coordination, spatial-temporal analysis, and driving mechanism research, with a view to making breakthroughs.

We introduce geospatial factors into the analysis of the spatial effects of urbanization coordination level, and construct an analysis model of the spatial effects of coordinated levels of urbanization from three aspects: Spatial correlation, spatial difference, and spatial pattern evolution. At the same time, referring to the relevant concepts and models of econometrics, the process of coordinated development of urbanization is regarded as the result of the input and output of different factors, and the driving force of the evolution of coordinated development of urbanization is analyzed through a spatial autoregressive model. The method constructed in this paper broadens and improves the relevant theoretical basis and research methods for the study of spatial effects of urbanization. On the one hand, through the measurement and analysis of the urbanization coordination level, it is helpful to strengthen the understanding of the current urbanization coordination level in China, and also to find problems and deficiencies in the process of urbanization. Then, the Chinese government can optimize the coordinated development of urbanization in the future. On the other hand, it helps to understand the differences in the level of urbanization coordination in different regions. In the later urbanization process, through reasonable adjustment of the distribution and scale of urbanization, and the tilt and support of policies, the development gap between different regions of China can be gradually reduced.

The rest of the paper is organized as follows. In Section 2, we introduce the study area. In Section 3, we introduce the methods used in this study. In Section 4, we take 286 cities in China as examples to analyze the coordination level and spatial effect of the quality and scale of urbanization. And then, we analyze the driving forces of the spatial-temporal pattern evolution of urbanization coordination level in China. In Section 5, we draw the main conclusions.

## 2. Study Area

**Figure 1:**
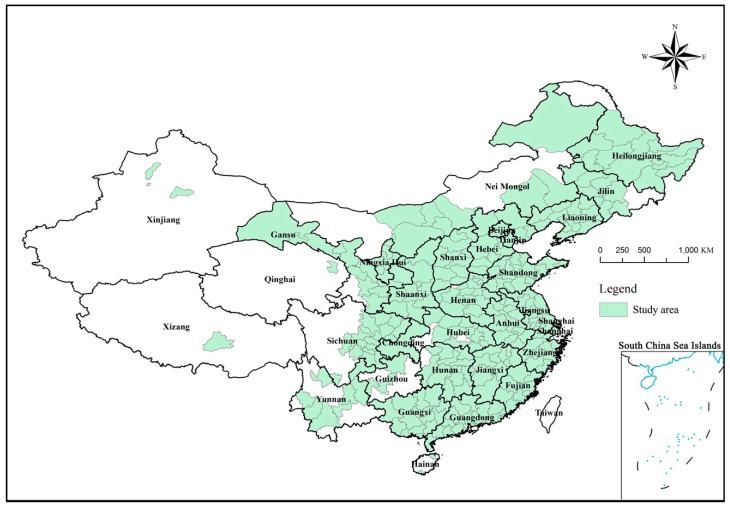
Study area.

Our study area includes 286 urban samples (The 286 samples include four municipalities and 282 prefecture-level cities in mainland China. In China, prefecture-level municipal governments are the most basic government that publish statistical data. In order to collect data, we choose the prefecture-level municipal administrative district as the study area) in mainland China (see as Figure 1). When collecting data in the paper, we found that there was a large number of missing data before 2005. Therefore, based on the availability of data and the span of research time, we finally selected the year from 2005 to 2015 as the study period.

## 3. Methods and Materials

### 3.1. The Methods for Urbanization Coordination Level

#### 3.1.1. The Method for Calculating Urbanization Coordination Level

Coordination is a benign interrelationship between systems or between system components. The level of coordination, or coordination degree, is a measure of the degree of harmony and consistency between systems or between elements within the system during the development process. It reflects the degree of benign coupling of the interactions between subsystems and the degree of coordination, and reflects the trend of the system from disorder to order [2,23]. In this article, we introduce this concept into the study of urbanization to describe the degree of coordination degree between the urbanization scale and urbanization quality in the process of urbanization (referred to as the urbanization coordination level). The specific model is as follows:

(1) $$Q_{i}=\sum_{i=1}^{n}W_{n}A_{n},$$

(2) $$S_{i}=\sum_{i=1}^{m}W_{m}B_{m},$$

(3) $$C_{i}=\frac{Q_{i}*S_{i}}{{\left(\alpha Q_{i}+\beta S_{i}\right)}^{2}},C\in \left[0,\;1\right],$$

(4) $$D_{i}=\sqrt{C_{i}*\left(Q_{i}+S_{i}\right)/2},$$

where *Qi* indicates the level of urbanization quality of the sample city *i*. *Si* represents the level of urbanization scale of the sample city *i*. *A* and *B* refer to the index factors of the quality and scale of urbanization, respectively. *n* represents the number of samples. *W* represents the weight value of each index. *Ci* represents the degree of coupling between the urbanization scale and urbanization quality. *α* and *β* represent weight parameters (In this article, the scale of urbanization and the quality of urbanization are two aspects that characterize the urbanization coordination level. The weight parameters of the two should be equal, so the values of α and β are 0.5). *Di* represents the level of urbanization coordination of city *i*.

#### 3.1.2. The Method for Determining Weight Value

To reduce the influence of human subjective factors on weight determination, we use the entropy weight method to determine the weight value of each evaluation index. Taking into account the influence of time variables, we improve the entropy weight method, the improved model is as follows:

The original data form the matrix *X*:

(5) $$X=\left[\begin{array}{ccc} X_{11} & \cdots & X_{IM}\\ \vdots & \ldots & \vdots \\ X_{N1} & \cdots & X_{NM} \end{array}\right],$$

By normalizing the raw data, we can get a new matrix *Y*:

(6) $$Y=\left[\begin{array}{ccc} Y_{11} & \cdots & Y_{IM}\\ \vdots & \ldots & \vdots \\ Y_{N1} & \cdots & Y_{NM} \end{array}\right],$$

Then, we can get the entropy value:

(7) $$e_{j}=-k\sum_{t}\sum_{i}P_{ij}\ln P_{ij},$$

(8) k=1/ln(t∗n),

(9) $$P_{ij}=Y_{ij}/\sum Y_{ij},$$

The weight value *W_j_* can be calculated based on the entropy value:

(10) $$P_{ij}=Y_{ij}/\sum Y_{ij},$$

where *X_ij_* is an element in matrix *X*, *t* is the number of years, *m* is the number of indices, *n* is the number of samples, and *e_j_* is the entropy value.

#### 3.1.3. Index System

The construction of the index system involves two aspects: The quality of urbanization and the scale of urbanization. (1) In terms of the quality of urbanization, based on the research of scholars at home and abroad, this article constructs an index system of the quality of urbanization from the four aspects of economic quality, social quality, ecological environment quality, and innovation quality [2,15,16,17,19,24]. (2) Considering the scale of urbanization, it is considered that not only the urban population changes in the process of urbanization, but also socio-economic and land. Based on the research of domestic and foreign scholars, this paper mainly constructs an urbanization scale index system from three aspects: Population scale, economic scale, and land scale [2,25,26]. Therefore, in constructing the indicator system of urbanization coordination level, this paper selects seven primary indicators and 38 secondary indicators from the two targets of urbanization quality and urbanization scale. The specific index system and related weights are shown in Table 1.

#### 3.1.4. Data

The data used in our paper are collected from the *China Statistical Yearbook* and *China Urban Statistical Yearbook*, which are published by the Statistic Bureaus of the Chinese central government and local government. Since the units and attributes of each index are different, the data cannot be directly calculated, and the data needs to be standardized. There are two types of attributes of the index system. Among them, if the index value is larger, the measured urbanization quality level value is larger, then such indices are positive index; on the contrary, if the index value is larger, the urbanization quality level value is smaller, such indices are called negative index.

The normalized formula for the positive index:

(11) $$A_{i}=\frac{a_{i}-\left\{a_{min}\right\}}{\left\{a_{max}\right\}-\left\{a_{min}\right\}}*100,$$

The normalized formula for the negative index:

(12) $$A_{i}=\frac{\left\{a_{max}\right\}-a_{i}}{\left\{a_{max}\right\}-\left\{a_{min}\right\}}*100,$$

$\left\{a_{max}\right\}\;$ and $\left\{a_{min}\right\}\;$ are the maximum and minimum values of each evaluation index in all years.

### 3.2. The Methods for Spatial-Temporal Features Analysis of Urbanization Coordination Level

The spatial elements have the functions of interdependence, mutual restriction, and mutual influence in geospatial space, which are the essential characteristics of geospatial phenomena and spatial processes. This spatial-temporal distribution of spatial elements contains two meanings that involve: (1) Both the distribution characteristics at the spatial level and the evolution characteristics on the time axis; and (2) both the correlation features and the differentiated features [27,28]. The analysis of the spatial effect of urbanization coordination level aims to use the spatial econometric model to analyze the spatial-temporal pattern of urbanization coordination level, and then explore the spatial agglomeration, radiation, or convergence effects and evolution trends of urbanization coordination level. Spatial effects can be divided into spatial correlations and spatial differences [28,29]. Spatial autocorrelation analysis is a method to describe the correlation between different variables. Its spatial distribution characteristics can be measured by two indicators: Global and local spatial autocorrelation. Spatial variability describes the variability that exists between different individuals, and is characterized by structural features in the form of model functions or parameters, and emphasizes that this difference is caused by spatial distribution or spatial structural characteristics [29].

#### 3.2.1. Spatial Autocorrelation Analysis Model

Spatial autocorrelation is the function of the interdependence, mutual restriction, and mutual influence of things and phenomena in space. It is an inherent property of things and phenomena and an essential feature of geographic space. It means that the observations at different positions are not independent in space and present a certain nonrandom spatial pattern [27,28]. Common spatial autocorrelation analysis models include global autocorrelation test and local autocorrelation test. By analyzing the global spatial autocorrelation of the urbanization coordination level of each sample in China, it is helpful to find the agglomeration effect of the urbanization coordination level. The global autocorrelation test is expressed by Moran ’s I index, and its formulas are:

(13) $$GM=\frac{\sum_{i=1}^{n}\sum_{i=1}^{n}w_{ij}\left(X_{i}-\bar{X}\right)\left(X_{j}-\bar{X}\right)}{S^{2}\sum_{i=1}^{n}\sum_{j=1}^{n}W_{ij}},$$

(14) $$S^{2}=\sum_{i=1}^{n}{\left(X_{i}-\bar{X}\right)}^{2}/n,$$

where *X_i_* is the observed value of city *i*, *X_j_* is the observed value of city *j*, and *W_ij_* is the spatial weight matrix. The value range of *GM* is generally between [−1, 1]. A value less than 0 indicates that city *i* and city *j* are negatively correlated, a value of 0 indicates that city *i* and city *j* are not related, and a value greater than 0 indicates that city *i* and city *j* are positively related.

Since global spatial autocorrelation analysis is difficult to detect regional spatial correlations that exist in different geographic locations, when it is necessary to reveal which spatial region contributes more to global spatial autocorrelation, local autocorrelation analysis must be performed. Local spatial autocorrelation analysis can be reflected by statistics of Local Moran’s I index [28]. Its formulas are:

(15) $$LM=\frac{\left(x_{i}-\bar{x}\right)}{S^{2}}\sum_{j=1}^{n}W_{ij}(x_{j}-\bar{x}),$$

(16) $$S^{2}=\sum_{i=1}^{n}{\left(X_{i}-\bar{X}\right)}^{2}/n,$$

When *LM* takes a value greater than 0, it indicates that the difference of urbanization coordination level between city *i* and its surrounding cities is less significant, that is, the target unit space are similar to those of neighboring units (there is “High-High” or “Low-Low ”agglomeration (“High-High” aggregation means that cities with a high level of urbanization coordination come together; “Low-Low” aggregation means that cities with a low level of urbanization coordination come together)); when the value is less than 0, it indicates that the difference between the urbanization coordination level of city *i* and its surrounding cities is significant, and the target space unit is not similar to the neighboring space unit (“High-Low” or “Low-High” aggregation (“High-Low” aggregation refers to the region where the level of urbanization coordination is high, but the surrounding cities are regions with a low level of urbanization coordination; “Low-High” aggregation refers to the area with low urbanization coordination cities, but surrounding cities are regions with a high level of urbanization coordination)).

#### 3.2.2. Spatial Difference Analysis Model

In the process of urbanization development, due to the different development bases and conditions of each sample city, differences are common in the process of urbanization development. In this paper, when analyzing the spatial difference in the coordination level between the urbanization quality and urbanization scale, by introducing the concept of the difference coefficient, the Urbanization Coefficient of Variation (UV) of urbanization coordination level is constructed to represent the actual situation of urbanization coordination level (divergence or convergence) [2,28]. The coefficient of variation is also called the coefficient of dispersion. It is the percentage of the standard deviation of a set of data and its mean, and it is a relative indicator of the degree of dispersion of the measured data. The specific models are:

(17) $$S=\sqrt{\sum_{i=1}^{n}{\left(Y_{i}-\bar{Y}\right)}^{2}/n},$$

(18) $$UV=S/\bar{Y},$$

where *Yi* represents the urbanization coordination level of city *i*. $\bar{Y}$ represents the average level of urbanization coordination among all sample cities in a year. *n* represents the number of samples. *S* represents the standard deviation of the urbanization coordination level in city *i*. The smaller the *UV* value, the smaller the difference in the urbanization coordination level between the samples, that is, it has a convergent feature. Meanwhile, the larger the *UV* value, the greater the difference in the urbanization coordination level between the samples, showing divergence feature.

#### 3.2.3. The Models for Pattern Evolution Analysis

Standard Deviational Ellipse Method (SDEM) was proposed by Prof. Lefever in 1926 [30,31]. The model works by calculating the standard distances in the *x* and *y* directions separately, using these two measurements to define an ellipse containing all the distributions of the elements and calculating the relevant parameters [30]. The long axis indicates the direction of data distribution, the short axis indicates the range and dispersion degree of the data distribution. The larger the value gap between the long axis and the short axis (i.e., the flatness) is, the more obvious the directivity of the data is. The center point represents the center of gravity of the spatial element layout. SDEM can identify the direction of a set of data and the trend of distribution. This study utilizes SDEM to analyze the spatial evolution of urbanization quality to understand the evolutionary trend of urbanization quality. The specific calculation process and formula of SDEM are as follows [30]:

Determine the center of the ellipse (*SDEx*, *SDEy*):

(19) $$SDE_{x}=\sqrt{\frac{\sum_{i=1}^{n}{\left(x_{i}-\bar{X}\right)}^{2}}{n}},$$

(20) $$SDE_{y}=\sqrt{\frac{\sum_{i=1}^{n}{\left(y_{i}-\bar{Y}\right)}^{2}}{n}},$$

where *xi* and *yi* represent the spatial coordinate position of each element, and $\bar{X}$ and $\bar{Y}$ represent the arithmetic mean center.

Determine the direction of the ellipse. The specific method is based on the *x*-axis, and the north direction is 0 degrees, which is obtained by rotating clockwise. The specific formula is as follows:

(21) $$\tan\theta =\frac{A+B}{C},$$

(22) $$A=\left(\sum_{i=1}^{n}{\tilde{x}}_{i}^{2}-\sum_{i=1}^{n}{\tilde{y}}_{i}^{2}\right),$$

(23) $$B=\sqrt{{\left(\sum_{i=1}^{n}{\tilde{x}}_{i}^{2}-\sum_{i=1}^{n}{\tilde{y}}_{i}^{2}\right)}^{2}+4{\left(\sum_{i=1}^{n}{\tilde{x}}_{i}{\tilde{y}}_{i}\right)}^{2}},$$

where ${\tilde{x}}_{i}$ and ${\tilde{y}}_{i}$ represent the difference between the mean center and each actual coordinate, and *θ* represents the direction angle.

Determine the length of the ellipse length axis *x* and *y*, as follows:

(24) $$\sigma_{x}=\sqrt{\frac{2\sum_{i=1}^{n}{\left({\tilde{x}}_{i}\cos\theta -{\tilde{y}}_{i}\sin\theta \right)}^{2}}{n}},$$

(25) $$\sigma_{y}=\sqrt{\frac{2\sum_{i=1}^{n}{\left({\tilde{x}}_{i}\sin\theta +{\tilde{y}}_{i}\cos\theta \right)}^{2}}{n}},$$

### 3.3. The Methods for Analyzing Driving Factors of Spatio-Temporal Pattern Evolution of Urbanization Quality and Urbanization Scale Coordination Level

The interrelationship between different spatial units will produce a geospatial dependency effect. In studying the influencing factors of the spatial-temporal pattern evolution of urbanization, it is necessary to consider the influence of geospatial correlation. On the one hand, it is necessary to consider the correlation and difference between samples at the same time. On the other hand, the evolution features of different samples on the time series scale must be considered [2,28]. Spatial econometric regression model based on panel data is an effective method to consider spatial correlation. The process of urbanization development is also a production process, that is, a process of obtaining an output level by combining certain inputs. The Douglas production function is the most commonly used mathematical model for analyzing and describing production relations in economics. This model represents the relationship between input and output under certain technical conditions over a certain period of time [32]. When dealing with actual economic problems, the production function is not only a correspondence between input and output, but also a constraint of production technology [32]. Based on this theory, a driving factor analysis model for the spatio-temporal pattern evolution of urbanization quality and urbanization scale coordination is constructed:

(26) $$Y=S*A_{1}{}^{\beta_{1}}*A_{2}{}^{\beta_{2}}*A_{3}{}^{\beta_{3}}*\cdots *A_{m}{}^{\beta_{n}},$$

On the basis of Equation (26), a spatial econometric model of urbanization coordination can be further constructed by adding spatial independent variables:

(27) $$lnY_{it}=\alpha +\beta_{1}ln{A_{1}}_{it}+\beta_{2}ln{A_{2}}_{it}+\beta_{3}ln{A_{3}}_{it}+\cdots +\beta_{n}ln{A_{m}}_{it}+\varepsilon_{it}.\;,$$

(28) $$\varepsilon \sim N\left(0,\sigma^{2}\right)$$

where *Y* is the interpreted variable. *S* represents a constant term. *A_1_* to Am indicate the factors that influence the spatial evolution of the coordination level of the urbanization quality and urbanization scale. α represents a constant term. *ε_it_* represents independent and identically distributed random error terms. Both *i* and *t* satisfy that the mean is zero and the variance is $\sigma^{2}$. *β1* to *βn* represent the parameters to be estimated for each factor.

## 4. Results and Discussion

### 4.1. Measuring Urbanization Coordination Level in China

**Figure 2:**
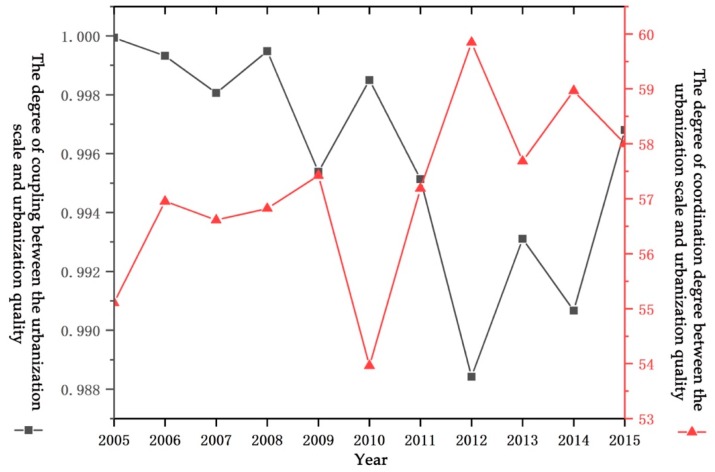
Overall urbanization coordination level in China from 2005 to 2015.

**Figure 3:**
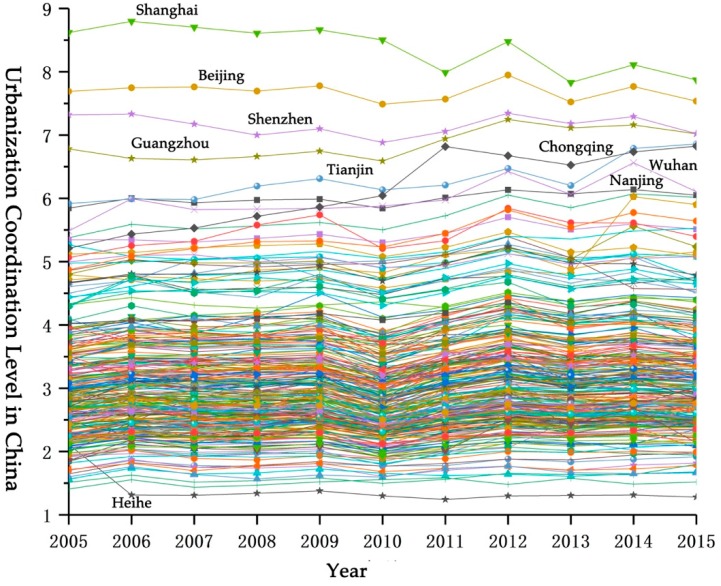
Urbanization coordination levels of sample cities from 2005 to 2015.

Based on the calculation of the urbanization quality and urbanization scale of each sample city in China, the coordination level of the urbanization quality and urbanization scale of each sample can be further obtained. At the national level, the overall level of urbanization coordination of China (see Figure 2) remained at a stable level from 2005 to 2015, and the overall fluctuation was small. In terms of changing trends, it shows a slowly rising trend. This is related to the shift in focus of urbanization development from a focus on scale and speed to a focus on the urbanization quality in recent years. At the same time, it is also affected by the urbanization development and transformation and upgrading policies introduced by the Chinese government. Judging from the changes in the urbanization coordination level of each sample city (see Figure 3), it shows the same trend as the overall level of urbanization coordination in China. From the statistical data: (1) The sample cities with the coordination level value between the interval (0, 4) accounted for the largest proportion, and the proportions in each year were above 80% (the average proportion reached 85%), which also illustrates the urbanization of most cities in the country. There is a serious inconsistency between the scale and the quality of urbanization, which is related to China’s long-term preference for scale development in urbanization and its policy preference for quality improvement; (2) sample cities with coordination level values in the interval [4, 5), The average proportion is about 8.7%; (3) the sample cities with a coordination level value between [5, 6) have an average proportion of 3.8%, mainly in the provincial capital cities in the central and eastern regions; (4) sample cities with a coordination degree range of [6, 7), with an average ratio of about 1.14%, mainly in Guangzhou, Nanjing, Tianjin, Chengdu, and other cities; (5) the sample cities with coordination degree between [7, 8) and [8, 10) belong to the development stage with good coordination of urbanization development, and the average proportion of the two stages is about 0.86% and 0.25%, mainly in Shanghai, Beijing, Shenzhen, and other megacities.

This confirms and explains the changes in the overall urbanization coordination level. At the same time, it can be seen that megacities and large cities have a higher level of urbanization coordination, which is inseparable from the large scale and quality of megacities and large cities. The coordination level in small and medium cities is relatively low.

### 4.2. Analysis on the Spatial-Temporal Features of the Coordination Level between Urbanization Quality and Urbanization Scale in China

#### 4.2.1. Spatial Correlation Analysis of the Coordination Level of Urbanization Quality and Urbanization Scale in China

**Figure 4:**
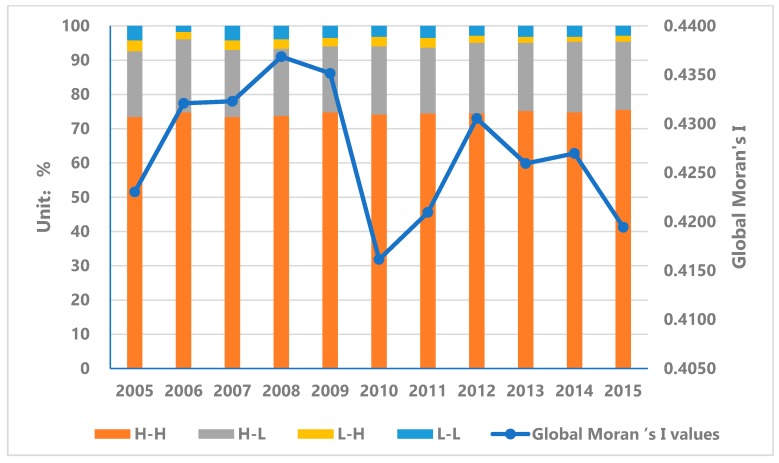
Global Moran’s I values of urbanization coordination level of sample cities in China from 2005 to 2015.

**Global Autocorrelation Analysis (Global Moran’s I value).** The results of the global autocorrelation analysis of the coordination levels of urbanization in 286 sample cities in China from 2005 to 2015 can be obtained through calculation. From the results (see Figure 4), the overall spatial correlation of urbanization coordination level is relatively high and the Global Moran’s I values are all above 0.415, which indicates that the sample cities in China show a more obvious positive correlation at the urbanization coordination level, that is, there are spatial agglomeration effects on a national scale. From different time periods: (1) From 2005 to 2009, the global autocorrelation level of the urbanization coordination level showed a slowly increasing correlation. Global Moran’s I value increased from 0.423 in 2005 to 0.435 in 2009, and reached the highest value of 0.437 in 2008. The increase reached 3.27%. (2) After 2009, the global autocorrelation index of the level of urbanization coordination dropped, and the value of Global Moran’s I decreased from 0.435 in 2009 to 0.416 in 2010. From 2010 to 2012, the overall level of urbanization coordination showed an upward trend, and the Moran’s I value increased from 0.416 in 2010 to 0.431 in 2012. (3) After 2012, the autocorrelation level of urbanization coordination level showed a downward trend again, and the value of Global Moran’s I decreased from 0.431 in 2012 to 0.419 in 2015.

**Figure 5:**
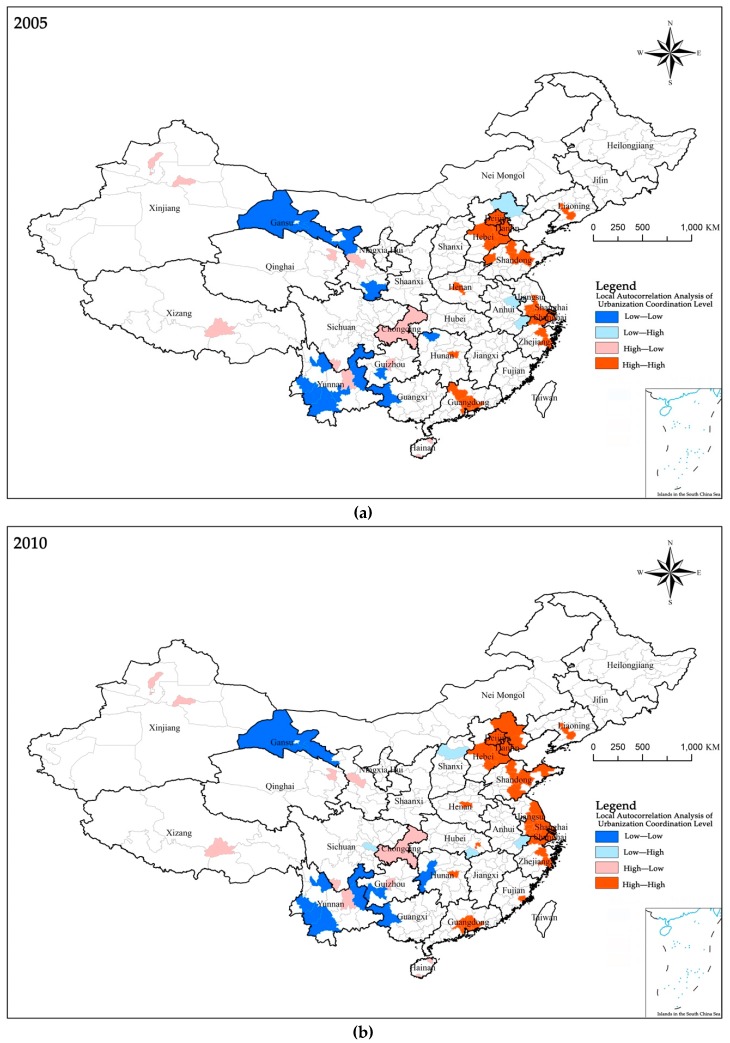

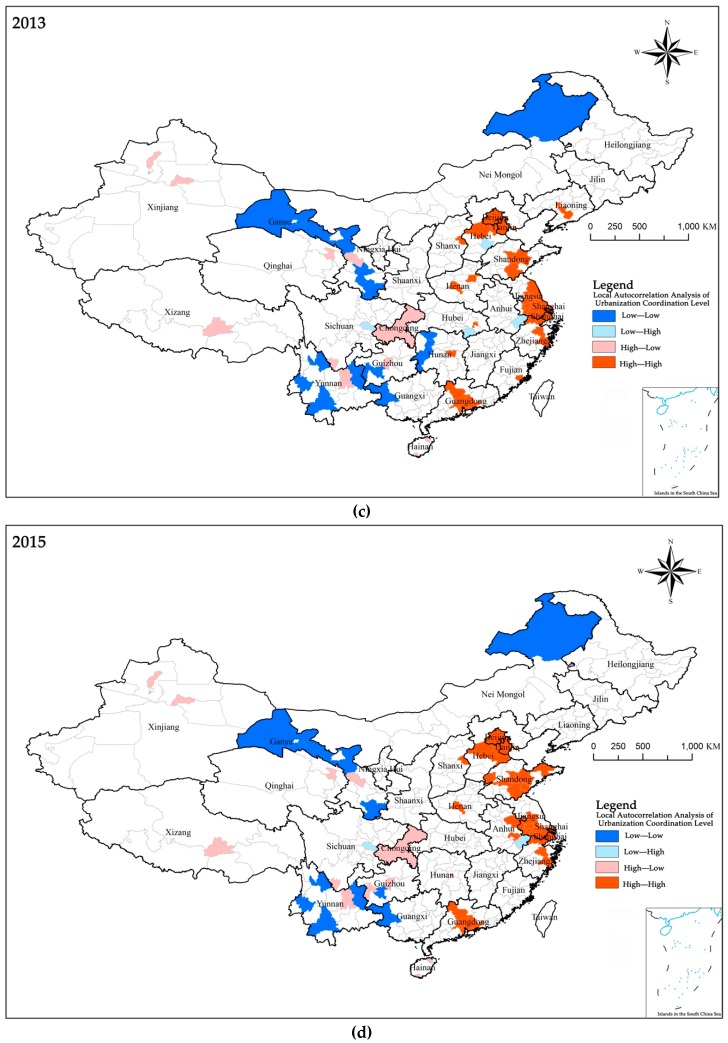
Local autocorrelation analysis of urbanization coordination level in China: (**a**) 2005; (**b**) 2010; (**c**) 2013 and (**d**) 2015.

**Local Autocorrelation Analysis** (**Local Moran’s I value**)**.** The global autocorrelation cannot reflect the spatial autocorrelation of local areas, that is, it cannot reflect whether there are radiation effects in local areas. Therefore, calculation and analysis of local spatial autocorrelation are needed. The results of the local autocorrelation analysis (Figure 5) show: (1) “High-High” aggregation areas are mainly concentrated in the Beijing-Tianjin-Hebei urban agglomeration, the Yangtze River Delta urban agglomeration, the Pearl River Delta urban agglomeration, the Shandong Peninsula, and the Central Plains urban agglomeration. This shows that the local agglomeration effect of the urbanization coordination level in these areas is gradually strengthened, that is, the radiation effect on the surrounding areas is significant. Among them, the Beijing-Tianjin-Hebei region also has a radiation effect on its surrounding major cities in Hebei Province, and the radiation effect has weakened after 2013. The urbanization coordination level of the Central Plains urban agglomeration is relatively weak and the change is relatively stable. The affected area is mainly concentrated in Henan Province. (2) The distribution of the “Low-Low” aggregation areas is relatively scattered. A small number of cities in the northwest, southwest, central, and northeast regions show low levels of radiation effects. The “Low-Low” aggregation areas in the Northwest are mainly concentrated in Gansu Province, and the “Low-Low” aggregation areas in the Southwest are mainly concentrated in Yunnan, Guangxi, Guizhou, and Sichuan. Among them, the low-level radiation effects are most obvious in Yunnan and Gansu provinces. This is mainly due to the low level of urbanization in these two areas, which leads to the spatial radiation effect of “Low-Low” concentration in the area. 3) In addition, the sample cities exhibiting “High-Low” aggregation and “Low-High” aggregation indicate that the urbanization coordination level of these regions has a discrete effect in space, that is, it has no radiation effect. The distribution of these areas is relatively scattered, and this part will not be analyzed in this article.

#### 4.2.2. Spatial Difference Analysis of the Coordination Level of Urbanization Quality and Urbanization Scale in China

**Figure 6:**
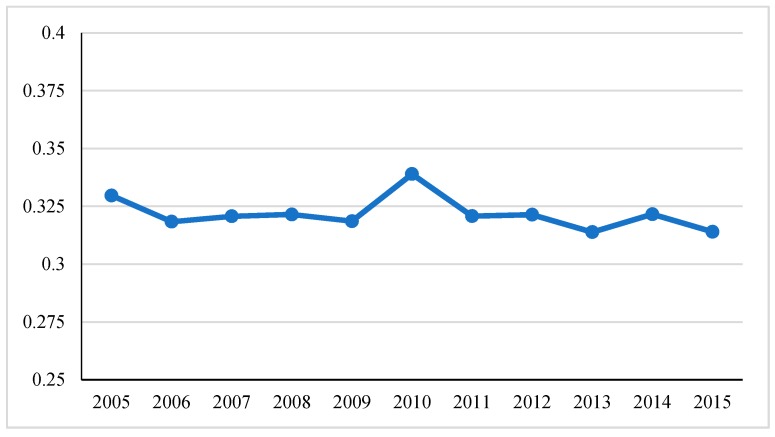
Index of spatial difference in urbanization coordination level from 2005 to 2015.

Through the analysis of the spatial autocorrelation model, we know that there is a significant spatial correlation between the sample cities and towns in China at the urbanization coordination level. However, whether this effect features are consistent with the actual situation requires further analysis. Through calculation, we can get the relevant difference index of China’s urbanization development from 2005 to 2015 (see Figure 6 for details). In general, except for the fluctuations in the values in 2010, the UV index of the urbanization coordination level remained in a stable range, which indicates that the convergence and divergence trends of the urbanization coordination level are not obvious. Among them, the UV index of the urbanization coordination level from 2005 to 2009 showed a slow downward trend. Comparing the global autocorrelation Moran’s I index during this period, it can be found that the autocorrelation in this period showed a slow upward trend and agglomeration effect. The strengthening reflects that the convergence rate of urbanization coordination on the UV index has increased. In 2010, the global autocorrelation Moran’s I index showed a turning point, which was reflected in the UV index of the coordinated level of urbanization as an increase in UV value. This shows that the convergence of urbanization coordination level has decreased and the divergence effect has accelerated. After 2010, the urbanization coordination level UV index remained within a stable level range, which was also verified on the global autocorrelation Moran’s I index. On the whole, comparing the changes in the spatial difference index UV and the global self-managed Moran’s I index at different stages of the urbanization coordination level, the two showed a certain negative correlation, that is, with the continuous improvement of spatial autocorrelation, the sample cities. The speed of convergence of urbanization coordination has been strengthened.

#### 4.2.3. Analysis for Spatial Pattern Evolution of the Coordination Level of Urbanization Quality and Urbanization Scale in China

**Figure 7:**
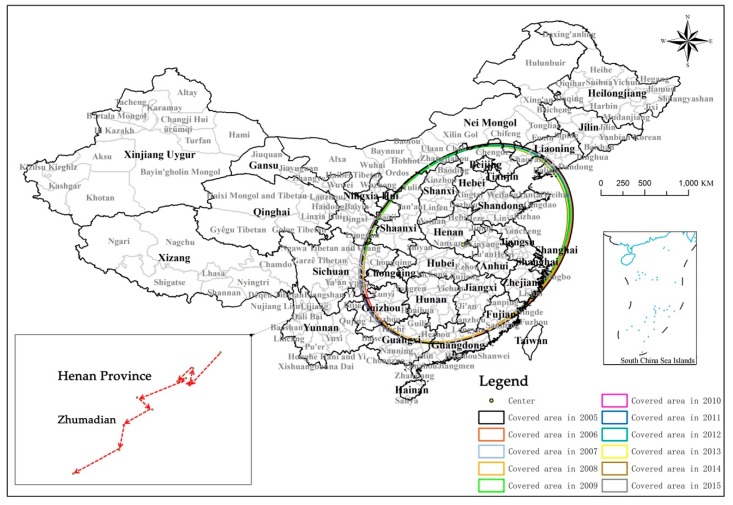
Evolution of the spatial distribution center and scope of urbanization coordination level in China from 2005 to 2015.

Judging from the standard deviation ellipse description statistics (Table 2) and spatial evolution layout (Figure 7) of the coordination levels of urbanization in various sample cities in China, from 2005 to 2015, the overall evolution pattern from northeast to southwest appeared. This shows that the vast central and western regions will become the main body of the improvement of China’s urbanization coordination.

Among them, the length of the major axis and minor axis of the ellipse is relatively small. The minor axis length was shortened from 7.833 in 2005 to 7.807 in 2015, and the major axis length was reduced from 11.178 in 2005 to 11.016 in 2015. It shows that on the national scale, the scope and dispersion of the spatial distribution of China’s urbanization coordination level are shrinking. The oblateness of the ellipse gradually becomes smaller, indicating that the directional characteristics of the coordination level of urbanization are becoming less and less obvious. This is because the difference in the coordination level of urbanization between the central and western regions is much smaller than the difference from the eastern region. Therefore, as the level of urbanization coordination evolved from northeast to southwest, the elliptical oblateness gradually became smaller.

From 2005 to 2015, the ellipse center showed a trend of deep development from northeast to southwest as a whole, and its distribution area was mainly concentrated in Zhumadian, Henan Province. The area covered by the ellipse has shrunk from 2,750,300 square kilometers in 2005 to 2,701,800 square kilometers in 2015. This shows that the distribution centers and coverage areas of urbanization coordination levels of sample cities in China have gradually spread to the southwest, but the changes are small, that is, the evolution of the spatial layout of urbanization coordination levels has changed little. In terms of spatial distribution, it is mainly concentrated in the central and eastern regions. In this region, the Yangtze River Delta urban agglomeration, the Beijing-Tianjin-Hebei urban agglomeration, and the Central Plains urban agglomeration, which have the highest degree of coordination of urbanization in China, are distributed. This is because the level of urbanization coordination in this region has been the highest in the region from 2005 to 2015, and the evolution characteristics of the spatial pattern also show a relatively unstable trend with a small change trend. The standard deviation ellipse is always distributed over this area.

At the same time, the vast southwestern, northwestern, and northeastern regions were low-value distribution areas of coordinated levels of urbanization from 2005 to 2015, and therefore remained outside the standard deviation ellipse coverage. In addition, although the Pearl River Delta region has always been a region with a higher level of urbanization coordination, it is not covered by the standard deviation ellipse. This is because the city but because of its widespread urbanization level of coordination around the lower value, the urbanization level of coordination from the Pearl River Delta region of 2005–2015 has been collecting area is low, that is, the level of development of the Pearl River Delta region of radiation on the surrounding the effect is not obvious.

### 4.3. Analysis of the Driving Forces of the Spatio-Temporal Pattern Evolution of Urbanization Coordination Level in China

#### 4.3.1. Discussion on the Dynamic Mechanism of the Coordinated Development of Urbanization

In the process of urbanization development, affected by various regional development basic conditions, industrial conditions, and institutional backgrounds, the spatial and temporal pattern of urbanization coordination levels will present different development trends. Among them, the main influencing factors are the driving factors of the spatial pattern evolution of urbanization coordination level. The analysis of the driving force of China’s urbanization coordination level is the premise and basis for understanding its mechanism and factors, and it is also an important reference and basis for promoting the healthy development of China’s new urbanization [33]. Referring to the perspectives of related scholars [2,16,33,34], we summarize the driving factors into five aspects: Administrative force, market force, outward force, endogenous force, and investment force. The driving mechanism of each driving factor on the evolution of the urbanization coordination level is mainly affected by industrial adjustment and reorganization, and allocation of funds and resources.

- ***Administrative Force.*** Administrative force is one of the main driving forces for the coordinated development of urbanization and the evolution of space-time patterns. Its role is mainly reflected by the following aspects: (1) The government guides the development of urbanization in a certain area through financial investment, industrial planning and layout, infrastructure construction, and planning of major functional areas. In particular, high-intensity infrastructure investment and major infrastructure construction have greatly enhanced the accessibility and closeness of the city to the outside world, improved the urban investment environment, and promoted the development of urban economy and urbanization. (2) The government transforms rural areas into urban areas directly through the adjustment of administrative force, especially administrative divisions, and directly promotes urbanization, or changes the rural population into urban population through the household registration system, which indirectly affects urbanization. When the population size and population density reach a certain level, it will cause tight urban living space, overburden public services and high urban operating costs, thereby reducing the quality of urbanization development. With the continuous expansion of population concentration, cities are forced to spread to the periphery, forming new residential areas and urban complexes, and triggering a new round of urban infrastructure investment. In general, the influence of administrative force on the coordinated development of urbanization has positive and negative effects, and specific analysis and judgment are needed based on the actual conditions of each region and the stage of development.
- ***Market Force.*** The market is a product of economic development, and its basic function is to play a fundamental role in regulating the allocation of resources in the development of urbanization, and to effectively allocate the production factors and geographical distribution in accordance with the laws of the market. The promotion of market forces on the development of urbanization is mainly reflected in the factors of production. Due to comparative advantages, production factors have been continuously concentrated in cities and nonagricultural industries, thereby promoting urbanization. As one of the main resources in the market, labor resources are most obviously regulated and allocated by the market. This is mainly reflected in the adjustment of the category structure ratio of employed persons and the diversification of employment subjects. In the process of urbanization, continuous improvement of the market mechanism has made it possible to freely transfer and flow all types of resources, thereby achieving the rapid development of China’s urbanization.
- ***Outward Force.*** The influence of external forces on the coordinated development of urbanization is mainly manifested in the effects of foreign investment and foreign trade on urbanization. Through the introduction of foreign capital and its advanced technologies, the conditions for capital formation will be improved, the changes in regional technology, trade, industrial structure, and employment structure will be promoted to improve the level of technology and management, thereby promoting the coordinated development of urbanization. The industrial and technological transfer in the process of economic globalization has provided a good opportunity for China’s economic development and further promoted the process of urbanization. While the development of foreign trade has improved China’s internationalization level and economic growth, it has also greatly promoted employment and absorbed a large amount of rural surplus labor. The industrial layout of foreign-funded enterprises has also driven local nonagriculturalization. Therefore, foreign investment and foreign trade are external motive forces for improving the development of urbanization.
- ***Endogenous force.*** Endogenous force refers to the intrinsic driving force of the coordinated development of a region, including the development of township and village enterprises, the suburbanization and migration of urban enterprises, and the suburbanization of industrial parks. The internal driving force for the development of urbanization depends on the rationality of the industrial structure. With the continuous advancement of industrial transfer and transformation and upgrading, urban centers mainly retain the tertiary industries such as financial services, while the secondary industries have migrated to the outer areas of cities and promoted the nonagriculturalization of rural areas to some extent. The nonagriculturalization of agricultural land has promoted the transformation of the primary industry into large-scale and industrialized operations. Therefore, it can be said that the industrial structure determines the regional economic growth mode and division of labor mode, and whether the industrial structure is reasonable or not is the internal driving force for the urbanization coordinated development.
- ***Investment Force.*** The influence of investment force on the urbanization coordinated development is mainly manifested in the form of capital investment. High-intensity capital investment will be transformed into a large number of fixed assets and public facilities, which will have a certain promotion and improvement effect on the scale and quality of urbanization, thereby helping to promote the urbanization coordinated level. At the same time, as the main body of investment in urban construction in China shifts from a traditional state-owned investment to a diversified investment body, the source of funding for urbanization and development has continued to widen, and the total amount of capital has continued to increase. The role of China is constantly strengthened, which in turn continues to promote the urbanization coordinated development.

#### 4.3.2. Empirical Analysis of the Driving Force of the Evolution of Urbanization Coordination Level in China

Based on the spatial panel regression econometric model, the MATLAB software was used to perform spatial econometric regression analysis on the driving factors of urbanization coordination levels in 286 cities from 2005 to 2015 (Table 3). From the regression results of OLS, it can be seen that each index passed the significance test of 1% significance level. Judging from the specific correlation coefficient, the industrial structure has the greatest impact on the level of urbanization coordination, and the external investment and market level have the second lowest impact. It is worth mentioning that the level of government administrative power and the level of urbanization coordination show a negative correlation with a high value based on the significance level. In the previous article, it has been proven that the coordination level of urbanization shows a significant spatial agglomeration effect in space. Therefore, on the basis of OLS analysis, this paper analyzes the driving force of the coordination level of urbanization with the help of Spatial Lag Model (SLM), Spatial Error Model (SEM), and Spatial Dubin Model (SDM).

It can be seen from the results in Table 3 that the three models of SLM, SEM, and SDM all passed the significance test of the corresponding significance level. From the log-likehood value, the SDM model has the largest value and is the better model. The R-squared values of SLM, SEM and SDM are 0.9837, 0.9806, and 0.9843, which are all close to 1, indicating that the model has a high degree of fit. It can be seen that the three spatial econometric models constructed basically meet the basic requirements for variable estimation, and can be used to analyze the main driving factors of urbanization coordination level. From the results of the three models of SLM, SEM, and SDM, each major parameter passed the significance test of the corresponding significance level. From the correlation coefficient of each parameter, except for the administrative force correlation coefficient is negative, the correlation coefficients of the other parameters are positive. This shows that in the process of urbanization coordinated development, the administrative force has a negative impact on the urbanization coordinated development, while market force, outward force, endogenous force, and investment force have a positive impact on the level of urbanization coordination. Among them, the endogenous force is the primary impact factor, followed by administrative and investment forces.

(1) From the correlation coefficients and results of administrative force (A), the evaluation coefficients of the three models all passed the significance level test. This shows that the government has negatively affected the level of urbanization coordination through fiscal expenditure, but the correlation level is low. This is inseparable from the improper positioning of the government role in the current urbanization development process. Although the government has promoted the expansion of the city through administrative measures, it has improved the level of infrastructure and public services to a certain extent. However, in the actual development of urbanization, many cities have heavy scale and light weight, which has reduced the quality of urbanization and the efficiency of resource utilization to a certain extent, and has caused various types of urban problems. The emergence and expansion of these negative effects offset the positive effects brought by the expansion of the city scale, and then a low negative correlation between administrative power and the level of urbanization coordination appeared. In addition, the w*lnA coefficient of the SDM model also passed the significance level test. This shows that the administrative power of a city will have a negative impact on the urbanization coordinated development in its neighboring cities. This is mainly due to the investment of local financial funds, which will attract and gather resources and elements from surrounding cities, especially large cities and megacities have a stronger impact. In general, the agglomeration effect produced by administrative power is greater than the diffusion effect, which can be corroborated by the results of the spatial correlation test.

(2) From the correlation coefficient and results of market power (M), the correlation coefficients of the three models are all above 0.006, and all have passed the significance test. This shows that market power has played a promoting role in the coordinated development of urbanization, that is, each increase of market power will promote the urbanization coordination level by 0.006 units. In the process of urbanization development, with the continuous improvement of the employment structure, the free transfer and movement of various resources has become possible, thereby improving the level of coordination of urbanization in China. From the result of w*lnM coefficient, the result failed the correlation test. This shows that the optimization of the industrial structure of a city only affects the level of local urbanization coordination, and the impact on the surrounding areas is not obvious.

(3) From the results of the correlation coefficient of outward force (O), the evaluation results of the three models all passed the significance level test. This shows that the outward force based on foreign direct investment has played a positive role in improving the coordination of urbanization. The increase in foreign direct investment can effectively improve the conditions for capital formation, drive changes in technology, trade, industrial structure and employment structure, improve technology and management levels, and promote coordinated development of urbanization. In addition, from the result of the w*lnO coefficient, the coefficient value has passed the significance test, which indicates that the increase of the outward force level of a city will also has a positive effect on the coordinated development of urbanization in neighboring areas. This also shows that in the process of urbanization development, keeping the market open will have a positive effect on the construction and development of urbanization in China.

(4) From the results of the correlation coefficient of the endogenous force (E), the correlation coefficients of the three models are all above 0.15, and all have passed the significance level test. This shows that the endogenous force has played a positive role in promoting the coordination of urbanization. This also verifies that in Section 4.1, the endogenous force represented by the industrial structure is the internal driving force for the coordinated development of a town or region, and a reasonable industrial structure can effectively promote the coordinated development of urbanization. However, from the w*lnE coefficient results of the SDM model, the correlation coefficients did not pass the significance test. This shows that a city’s endogenous power mainly plays a positive role in promoting the coordinated development of local urbanization, but has no significant impact on its surrounding cities.

(5) From the results of the correlation coefficient of investment force (I), the evaluation coefficients of the three models have passed the significance test, which indicates that the investment force plays a positive role in promoting coordinated urbanization. The development of urbanization must be based on infrastructure. The current development of urbanization in China emphasizes improving the quality of life of residents, which must also rely on good infrastructure. From this point, the improvement of the investment level will promote the promotion of urbanization coordination. At the same time, from the result of w*lnI coefficient, the correlation coefficient value passed the significance test. This shows that a city’s good infrastructure can also bring positive effects to the coordinated development of urbanization in surrounding cities, that is, there is a certain spillover effect of the level of investment power.

## 5. Conclusions

The quality of urbanization and the scale of urbanization are the two most important aspects in the process of urbanization. The measurement and analysis of the level of coordination between the two aspects will help to recognize and understand the differences in the level of urbanization coordination between different regions in China. This will help promote the healthy development of urbanization and promote urban–rural integration in the country. In this article, we first built an evaluation index system based on the quality and scale of urbanization, and built a spatial-temporal analysis model of the coordination level of urbanization from three aspects: Spatial correlation, spatial difference, and spatial pattern evolution. Finally, by introducing the related methods of spatial econometric regression, a driving factor model of the spatiotemporal pattern evolution of urbanization quality and urbanization scale coordination level was constructed. Based on a theoretical model, the article makes an empirical analysis of the level of urbanization coordination of 286 sample cities and towns in China from 2005 to 2015. The results show that: (1) The coordination level between the quality of urbanization and the scale of urbanization shows a strong spatial correlation in space, which is consistent with the actual development status; (2) the coordination level of urbanization is uniform in the evolution of the spatial and temporal pattern. It shows the evolution trend from northeast to southwest, but the change is small. (3) The changes in the spatial and temporal pattern of the coordination level of urbanization are affected by different driving forces, of which endogenous force is the primary factor, followed by administrative power and investment power.

On the one hand, the theories and methods proposed in this article help strengthen the understanding of the current level of urbanization coordination in China. It is helpful to find problems and deficiencies, to promote strengths and avoid weaknesses, to optimize the coordinated development of urbanization in the future urbanization development process. On the other hand, it helps to understand the differences in the level of urbanization coordination in different regions. In the later urbanization development process, through reasonable adjustment of the distribution and scale of urbanization, as well as through the tilt and support of policies, to gradually narrow the development gap between different regions.

## Figures and Tables

**Table 1:** Index system of coordination level between urbanization quality and urbanization scale.

Target Index	First Grade Index	Basic Grade Index	Attribute ^1^	Weight Value
Urbanization Quality	Economic Quality	Per capita GDP (RMB)	Positive	0.0174
		Per capita disposable income of urban residents (RMB)	Positive	0.0130
		Per capita local fiscal revenue (RMB)	Positive	0.0334
		Urban per capita consumption expenditure (RMB)	Positive	0.0225
		The total retail sales of social consumer goods (RMB)	Positive	0.0889
		Total investment in social fixed assets (RMB)	Positive	0.0293
		Per capita nonagricultural value added value of urban residents (RMB)	Positive	0.0278
		Actual foreign direct investment (US dollar)	Positive	0.0597
		Total imports and exports (%)	Positive	0.0488
	Social Quality	Per capita urban road area (km^2^)	Positive	0.0150
		Number of buses per 10,000 people (One in 10,000)	Positive	0.0254
		Number of doctors per 10,000 people (One in 10,000 people)	Positive	0.0100
		Number of hospital beds per 10,000 people (One in 10,000 people)	Positive	0.0091
		Number books in the public library per 10,000 people (One in 10,000 people)	Positive	0.0341
		Number of university students (Per person)	Positive	0.1596
		Number of Internet users (Per household)	Positive	0.1596
		Urban Engel coefficient (%)	Negative	0.0058
		Urban employees account for the proportion of the total population of the region (%)	Positive	0.0258
		Per capita housing area (km^2^)	Positive	0.0080
		Education expenditure accounts for the proportion of local fiscal expenditure (%)	Positive	0.0061
	Environmental Quality	Comprehensive utilization rate of industrial solid waste (%)	Positive	0.0212
		Harmless treatment rate of domestic garbage (%)	Positive	0.0081
		Sewage treatment rate (%)	Positive	0.0085
		Per capita green area (km^2^)	Positive	0.0289
		Green area coverage in built-up areas (%)	Positive	0.0080
	Innovation Quality	Science and technology expenditures account for the proportion of fiscal expenditure (%)	Positive	0.0232
		Number of patent applications (Per piece)	Positive	0.0753
		The number of scientific research and technical personnel population accounts for the total employed population (%)	Positive	0.0276
Urbanization Scale	Population Scale	Urban population	Positive	0.1336
		Proportion of urban population in total population (%)	Positive	0.0694
		Proportion of nonagricultural employment (%)	Positive	0.0015
		Urban population density (People Per Unit Area)	Positive	0.0731
	Economic Scale	Urban GDP (RMB)	Positive	0.1582
		The proportion of the output value of the secondary and tertiary industries in urban GDP (%)	Positive	0.0048
	Land Scale	Total area of urban built-up area (hm^2^)	Positive	0.1434
		Proportion of built-up area to total urban area (%)	Positive	0.1261
		Urban construction land area (km^2^)	Positive	0.1654
		Proportion of urban construction land in urban area (%)	Positive	0.1245

^1^ In this article, the attributes of indicators are mainly divided into two categories: Positive indicators and negative indicators. Positive indicators are indicators that have a positive impact on the final evaluation result, that is, the larger the value of the indicator, the larger the evaluation result. Negative indicators are indicators that have a negative impact on the final evaluation result, that is, the larger the index value, the smaller the evaluation result.

**Table 2:** Ellipse description parameters of standard deviation of urbanization coordination level from 2005 to 2015.

Year	Center		Length		Flatness
	CenterX(E) ^1^	CenterY(N) ^2^	XStdDist ^3^	YStdDist ^4^	
2005	114.53	32.96	7.83	11.18	3.34
2006	114.49	32.93	7.81	11.11	3.30
2007	114.49	32.93	7.81	11.10	3.29
2008	114.50	32.94	7.80	11.08	3.28
2009	114.48	32.93	7.79	11.07	3.28
2010	114.49	32.93	7.84	11.08	3.24
2011	114.43	32.91	7.83	11.08	3.25
2012	114.45	32.90	7.80	11.05	3.26
2013	114.42	32.88	7.81	11.05	3.24
2014	114.41	32.85	7.81	11.04	3.22
2015	114.36	32.83	7.81	11.02	3.21

^1^ In this article, CenterX(E) refers to longitude. ^2^ CenterY(N) refers to latitude.^3^ XStdDist represents the short axis of the ellipse.^4^ YStdDist epresents the long axis of the ellipse.

**Table 3:** Spatial regression model of driving forces for urbanization coordination level.

Variable Parameter	OLS	SLM	SEM	SDM
ln A	−0.0878 ***	−0.0151 ***	−0.0040 *	−0.0177 ***
	(−12.1918)	(−6.2349)	(−1.3819)	(−4.8274)
ln M	0.0368 ***	0.0070 **	0.0084 ***	0.0063 **
	(4.0674)	(2.2941)	(2.7400)	(2.0696)
ln O	0.0820 ***	0.0025 ***	0.0026***	0.0022 **
	(49.6531)	(2.6667)	(2.7327)	(2.2908)
ln E	0.9700 ***	0.1599 ***	0.1755***	0.1783 ***
	(32.4133)	(12.6249)	(12.1024)	(12.1011)
ln I	0.0178 ***	0.0277 ***	0.0240 ***	0.0139 ***
	(2.6560)	(13.1651)	(10.1574)	(5.6941)
W*ln A	——	——	——	−0.0294 ***
				(−5.8693)
W*ln M	——	——	——	−0.0002
				(−0.0256)
W*ln O	——	——	——	0.0049 **
				(2.5043)
W*ln E	——	——	——	0.0301 *
				(1.3753)
W *ln I	——	——	——	0.0454 ***
				(11.3538)
W * ln Y	——	0.4130 ***	——	0.2820 ***
		(13.1118)		(7.9210)
R-Squared	0.6679	0.9837	0.9806	0.9843
sigma^2^	0.0276	0.0014	0.0014	0.0013
Log-Likehood	1183.50	5874.2049	5815.6384	5956.6271

Note: The values in parentheses represent the t-value statistics of the corresponding variables. ***, **, and * indicate that the variables are significant at levels of 1%, 5%, and 10%.
